# Combined Organic Photovoltaic Cells and Ultra Low Power CMOS Circuit for Indoor Light Energy Harvesting

**DOI:** 10.3390/s19081803

**Published:** 2019-04-15

**Authors:** Duarte Batista, Luis Bica Oliveira, Nuno Paulino, Carlos Carvalho, João P. Oliveira, Joana Farinhas, Ana Charas, Pedro Mendonça dos Santos

**Affiliations:** 1 CTS-UNINOVA, Instituto de Desenvolvimento de Novas Tecnologias, 2829-517 Caparica, Portugal; da.batista@campus.fct.unl.pt (D.B.); l.oliveira@fct.unl.pt (L.B.O.); nunop@uninova.pt (N.P.); cfc@cedet.isel.ipl.pt (C.C.); 2 Department of Electrical Engineering, Faculty of Sciences and Technology (FCT), Univ. Nova de Lisboa (UNL), 1099-085 Lisboa, Portugal; 3 Instituto de Telecomunicações, Av. Rovisco Pais 1, 1049-001 Lisboa, Portugal; joana.farinhas@lx.it.pt (J.F.); ana.charas@lx.it.pt (A.C.); pms@lx.it.pt (P.M.d.S.); 4 ADEETC, Instituto Superior de Engenharia de Lisboa (IPL-ISEL), CEDET, 1959-007 Lisboa, Portugal; 5 Academia Militar (AM), Instituto Universitário Militar (IUM), DCEE, Campus da Amadora, 2720-113 Amadora, Portugal

**Keywords:** indoor light harvesting, organic photovoltaic cells, integrated circuit, CMOS technology

## Abstract

This paper describes an energy harvesting system composed of an organic photovoltaic cell (OPV) connected to a DC–DC converter, designed in a 130 nm Complementary Metal-Oxide-Semiconductor (CMOS) technology, with a quasi- maximum power point tracking (MPPT) algorithm to maximize the system efficiency, for indoor applications. OPVs are an emerging technology with potential for low cost indoor light energy harvesting. The OPV current-voltage curves (I-V) under an irradiance of solar simulator Oriel Sol 3A, at room temperature, are obtained and an accurate electrical model is derived. The energy harvesting system is subjected to four different indoor light sources: 35 W halogen, 3.5 W LED, 5 W LED, and 7 W LED, positioned at three different heights (0.45 m, 0.26 m, and 0.11 m), to evaluate the potential of the system for indoor applications. The measurements showed maximum efficiencies of 60% for 35 W halogen and 45% for 7 W LED at the highest distance (0.45 m) and between 60% (5 W LED) and 70% (35 W halogen), at the shorter distance (0.11 m). Under irradiation, the integrated CMOS circuit presented a maximum efficiency of 75.76%, which is, to the best of the authors’ knowledge, the best reported power management unit (PMU) energy system using organic photovoltaic cells.

## 1. Introduction

**Figure 1:**
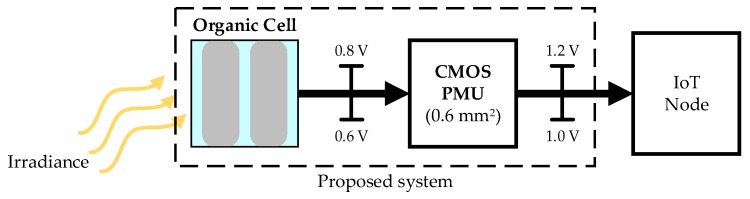
Indoor application of a PV cell.

The downscaling of integrated circuit technology, especially CMOS, is pushing the limits of miniaturization and portability. The availability of ultra-low power (µW) and complex monolithic circuits in CMOS nanotechnology opens new research on Internet of Things (IoT) solutions for a wide range of applications [1,2,3,4,5,6,7,8,9,10,11,12,13,14,15,16,17,18], including sensing, data processing, and communication. The power requirement for these systems depends on the application. Although these ‘smart’ sensors are typically battery powered [19], the use of harvesting techniques to obtain energy from different sources, such as ambient light, vibration/motion of human body parts, temperature differences, and Radio Frequency (RF) electromagnetic radiation, can improve battery lifetime and energy autonomy. Harvesting from the sun or ambient light is the most effective way to power a sensor node, with power densities in the range of tens of mW/cm^2^ [20,21]. Light energy harvesting (Figure 1) depends upon the ambient light to be harvested, which may be a mix between natural light coming from the outside, and artificial light provided by overhead lamps or luminaries.

A previous work [21] that reported on indoor light availability, in which the artificial light was generated by fluorescent light tubes, showed that when placing the harvester very close to overhead lamps, it was possible to achieve an irradiance of 10 W/m^2^. A more in-depth study was carried out in [22], in which the light availability inside an office was assessed for different times of the day, encompassing various ways to place and orient a harvester, either giving more emphasis to natural light, artificial light, or a combination of both. The worst-case irradiance during daytime, for solar radiation inside the room, had the value of 130 mW/m^2^. In addition, when using artificial light only, at night time, the worst-case measurement yielded a value of 730 mW/m^2^. The artificial light was provided by two fluorescent light tubes, each with a power of 36 W [22].

More recent studies have mainly focused on the use of flexible photovoltaic films of various natures, under LED lighting [23], taking into consideration that the typical irradiance amounts of 66 mW/m^2^, down to 7 mW/m^2^, are enough for the tested photovoltaic (PV) harvesting modules to operate favorably. The operating spectrum of the LED devices considered in [23] are very similar to those used in the present work.

In this work, an organic photovoltaic cell is used as the harvesting device. OPVs is an emerging technology based on heterojunctions of organic semiconductors (organic compounds, polymers, carbon-nanoparticles) [24,25,26]. These compounds can have very different chemical structures and properties. The solubility in common solvents is important because it allows the active/organic layer of the devices to be processed from solutions, i.e., as inks, by low cost deposition methods (e.g., spin coating, ink-jet printing, or roll-to-roll printing) over flexible substrates (plastics, e.g., PET, polyethylene terephtalate). The unique characteristics of OPVs, such as being lightweight and flexible, combined with their performance under diffuse light, allows for applications that more conventional technologies, such as crystalline silicon-based cells, cannot be easily adapted to, despite their higher efficiencies. State-of-the-art OPVs have already achieved power density levels of 14 mW/cm^2^ under simulated solar light of 100 mW/cm^2^ in laboratory prototypes, using glass substrates [27].

Typically, harvesting devices are connected to a power management unit based on a separated DC–DC converter. The harvested source is typically a thermoelectric generator (TEG) and the DC–DC conversion is mostly based on switched inductive converters (usually boost type), with battery assisted, mechanical, capacitor pre-charge, RF kick-start, or even step-up transformers power-up schemes [28,29,30,31,32]. Fully electric power-up solutions from a few tens of mV can be a CMOS Integrated Circuit (IC) and external high-value energy storage components (values of external inductances and capacitances can be as high as 1 mH and 100 μF, respectively) on a separate Printed Circuit Board (PCB) [33,34,35]. The DC–DC converters have been designed for relatively small input voltage variations (few tens to few hundreds of mV), due to the TEG harvesting output range. However, in contrast with TEGs, harvesting from PV cells requires a converter for a wider range of input voltages (100 mV < V_OC_ < 1 V). There are few reported works on step-up conversion of a PV cell output voltage, [36,37,38,39,40].

**Figure 2:**
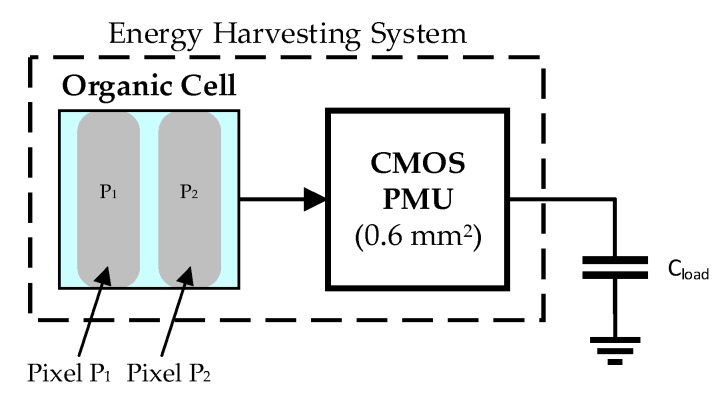
Proposed energy harvesting system.

Battery powered systems usually include a power management unit (PMU) to achieve a fixed regulated voltage with variable battery charge levels [41,42,43,44,45,46,47]. The architecture of the system proposed in this work is new with respect to state-of-the-art systems, as it leads to a small size (<1 cm^2^) and an energy self-sustainable device (Figure 2), suitable for portable applications.

The main goal of this paper is the development of a batteryless ultralow-power energy supply system, combing organic photovoltaic cell and a PMU (an integrated CMOS circuit) for indoor applications.

## 2. Characterization of a Photovoltaic Cell

### 2.1. OPV Fabrication and Characteristics

The OPV devices were prepared on indium tin oxide (ITO), (100 nm thick)-coated glass substrates previously cleaned sequentially with distilled water and a non-ionic detergent, distilled water, acetone, and isopropyl alcohol under ultrasounds.

The OPV cell consisted of a multilayer device with the structure: Glass/ITO/PEDOT:PSS/PffBT4T-2OD:PC_60_BM/LiF/Al cells, where:

- ITO is a conducting oxide;
- PEDOT:PSS is the conducting polymer Poly(3,4-ethylenedioxythiophene): poly(styrenesulfonate) (ca. 40 nm thick) acting as a hole transport layer;
- LiF/Al is the top metallic electrode, which is thermally evaporated on top of the active layer (LiF is 1.5 nm thick and Al is ca. 100 nm thick).

The organic/active layer was a mixture of PffBT4T-2OD and PC_60_BM:

- PffBT4T-2OD is poly[(5,6-difluoro-2,1,3-benzothiadiazol-4,7-diyl)-*alt*-(3,3′′′-di(2-octyldodecyl)-2,2′;5′,2′′;5′′,2′′′-quaterthiophen-5,5′′′-diyl)];
- PC_60_BM is [6,6]-Phenyl C61 butyric acid methyl ester [48,49].

**Figure 3:**
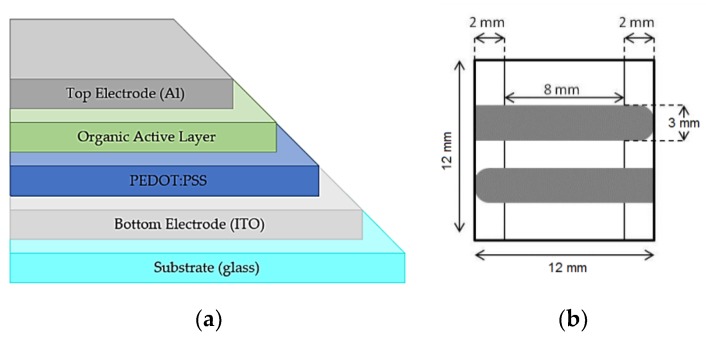
(**a**) Organic photovoltaic cell (OPV) structure of the organic PV cell used in this work: Glass/ITO/PEDOT:PSS/:PC60BM/LiF/Al; (**b**) active area 0.24 cm^2^.

The device architecture is shown in Figure 3:

### 2.2. OPV Electrical Model (I-V Characterization)

A photovoltaic cell can be characterized from its current-voltage curves (I-V), obtained in two different light conditions: Dark and standard solar conditions, i.e., under AM1.5G light with an irradiance of 100 mW/cm^2^.

**Figure 4:**
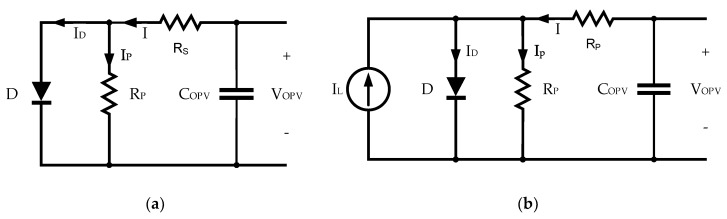
Equivalent circuit of a photovoltaic cell: (**a**) In dark conditions; (**b**) at standard irradiance conditions (1000 W/m^2^).

The equivalent circuit of the cell, as shown in Figure 4a, can be obtained from the curve in the dark. It is described by a combination of a junction diode *D* and two resistors (*R_s_* and *R_p_*). The resistors *R_s_* and *R_p_* are associated with losses and leakage currents, respectively.

The cells were fabricated with an absorbing layer composed of a bulk heterojunction of organic semiconductors, where the p-type semiconductor is a polymer, and the n-type semiconductor is a fullerene. When the light radiation struck the active layer, through the glass transparent electrode, a flow of charges was generated, which can be collected near the electrodes area. In this way, the diode D in the equivalent circuit diagram, allowed to characterize the p-n junction. The equation of *I_d_* running through the diode is depicted in (1), where *I_s_* is the saturation current of the diode, *n* is the diode factor, *V_D_* is the voltage at the terminals of the diode and *V_T_* is the thermal voltage. It should be noted that *V_D_* = *Vout* − *I* × *R_s_*.

(1) $$I_{d}{=I}_{s}\left(e^{\frac{V_{D}}{{nV}_{T}}}-1\right)$$

The thermal voltage is defined from Equation (2) where *k* represents Boltzmann’s constant, *T* the cell temperature and *q* the electron charge.

(2) $$V_{T}=\frac{kT}{q}$$

The resistors represent a decrease in the efficiency of the photovoltaic cell. It is assumed that the current delivered to the load in the dark is (3).

(3) $$I_{out}{=I}_{s}\left(e^{\frac{V_{out}{-IR}_{s}}{{nV}_{T}}}-1\right)+\frac{V_{out}{-IR}_{s}}{R_{p}}$$

The cell generated an *I_L_* current as a result of the incident radiation, as shown in Figure 4. The current at the terminals of the photovoltaic cell, *I_out_*, under standard irradiance conditions, is

(4) $${I=I}_{s}\left(e^{\frac{V_{out}{-IR}_{s}}{{nV}_{T}}}-1\right)+\frac{V_{out}{-IR}_{s}}{R_{p}}{-I}_{L}$$

This equation shows that the efficiency of the cell is dependent on the two resistors and the diode parameters in the equivalent circuit. To enhance the efficiency of the PV cells, the series resistance *R_s_* should be close to zero and the parallel resistance *R_p_* should be as large as possible, ideally infinite.

The Fill Factor (*FF*) is figure-of-merit used to specify the quality of the PV cell. Considering that the voltage provided by the cell varies between zero and the open-circuit voltage *V_oc_*, and that the current supplied varies between zero and the value of the short-circuit current *I_sc_*, there is a point on the characteristic curve of the cell, where the output power is maximum, *P_max_*, i.e., this voltage/current product reaches its maximum value, with the correspondent values of current (*I_mp_*) and voltage (*V_mp_*). Thus, the fill factor of the photovoltaic cell, is defined as the ratio between *P_max_* and the product of the open-circuit voltage *V_oc_* and the short-circuit current *I_sc_*,

(5) $$FF=\frac{P_{max}}{V_{oc}I_{sc}}=\frac{V_{mp}I_{mp}}{V_{oc}I_{sc}}$$

It is desirable to have a *FF* close to 1. The capacitance of a photovoltaic cell can be represented by a capacitor in parallel with the output of the cell [50],

(6) $$C_{OPV}=\frac{\varepsilon_{0}K_{s}A}{d}$$

where *ε*_0_ corresponds to the permittivity in vacuum, *K_s_* is the dielectric constant of the semiconductor, the active area of the cell is given by *A* and the semiconductor thickness is defined by *d*. The current–voltage (I-V) characteristics are measured in an inert atmosphere (N_2_) using a Keithley K2400 source-meter unit. The curves under illumination are measured with the solar simulator Oriel Sol 3A, 69920, Newport.

The power conversion efficiency (PCE) of the cells can be calculated from their I-V curves under illumination, using the open-circuit voltage (*Voc*), the short-circuit current (*Isc*), and the *FF*.

(7) $$\eta =\frac{P_{out}}{P_{in}}=\frac{I_{sc}\cdot V_{oc}\cdot FF}{P_{in}}$$

where *P_out_* is the maximum power generated by the device, *P_in_* represents the power of the incident light (provided by the solar simulator lamp), and *FF* is defined as in (5).

For each level of irradiance, each PV cell has a specific behaviour, with different values of *V_oc_* and *V_mp_*. These two voltages are related to each other by a factor *k*, which can be defined as

(8) $$k=\frac{V_{mp}}{V_{oc}}.$$

This characteristic will be exploited in the maximum power point tracking (MPPT) method that is selected for this application, considering the specific characteristics of the OPV cells that serve as harvesters in this work.

### 2.3. Experimental Characterization of the OPV

From the electrical point of view, the two terminals coming from the pixels implement the negative poles of each OPV while the terminal of the ITO corresponds to the positive terminal, which is common to the two pixels of the substrate. It should also be noted that the active area of each pixel was 0.24 cm^2^.

**Figure 5:**
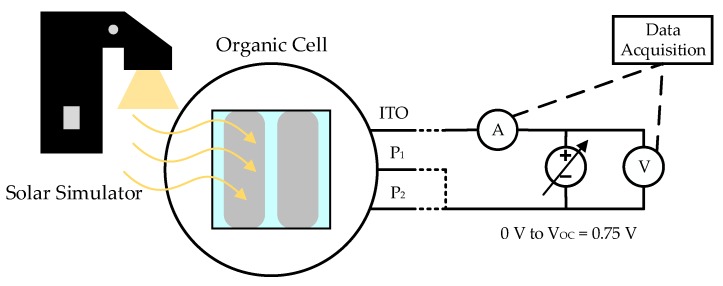
Measure setup for OPV characterization.

The measurement setup, in which the I-V curves were obtained (by averaging of the two pixels substrate), is shown in Figure 5. A light source was required (solar simulator) and an output variable voltage source, in which the output current was measured for different output voltage values using a data acquisition system.

**Figure 6:**
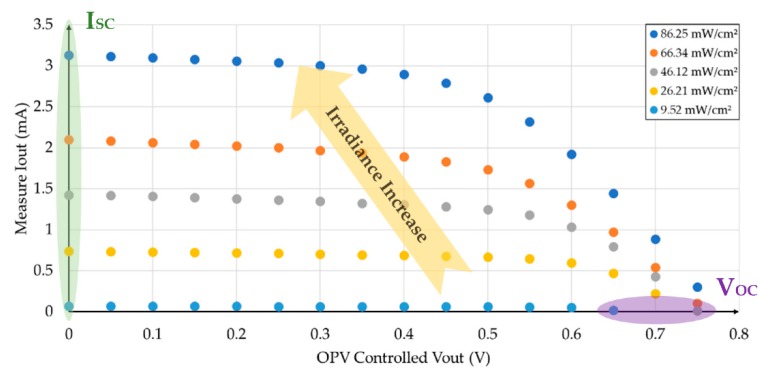
Measured I-V characteristics for different lighting levels in the OPV substrate.

**Figure 7:**
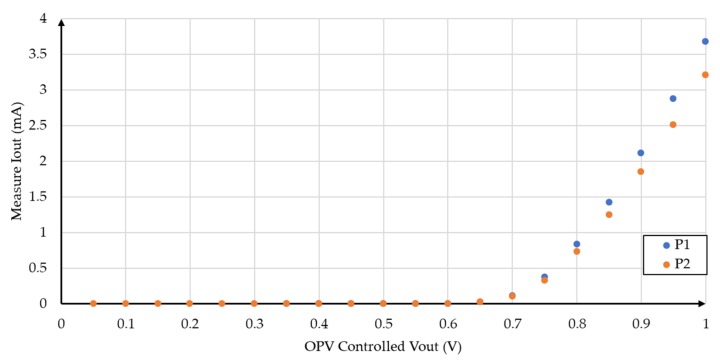
Measured I-V characteristics in dark conditions for the two pixels in the OPV substrate.

Four different incident radiation densities were considered, while keeping the sample-source distance of 19.5 cm, as shown in Figure 6. Figure 7 shows the measured I-V characteristic of an organic photovoltaic cell in dark condition.

**Figure 8:**
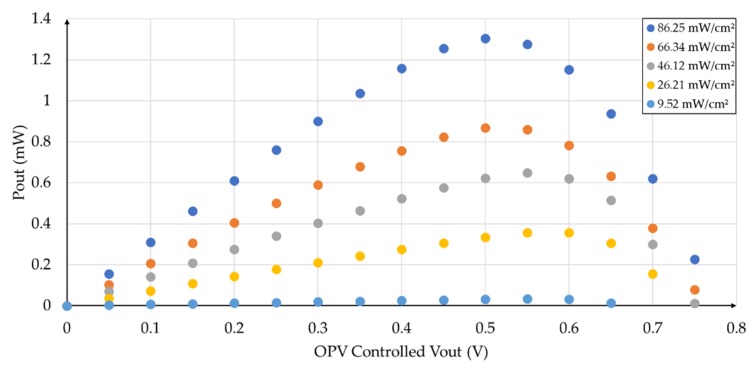
Experimental power curves of an OPV for different measured lighting levels.

Considering that the main function of a photovoltaic cell is to provide power to a load, the OPV power curves were obtained for the different levels of incident radiation (based on the I-V curves of Figure 6), as shown in Figure 8.

**Figure 9:**
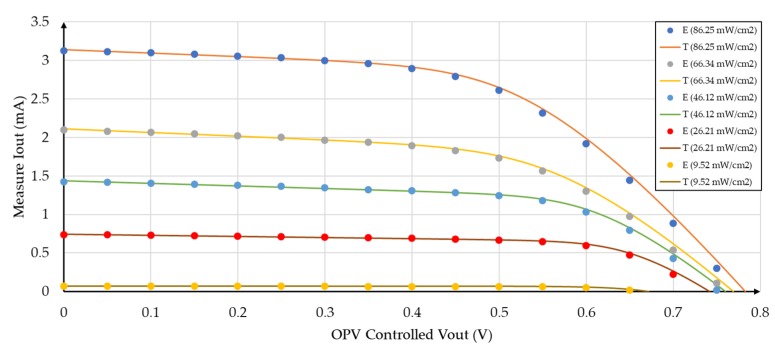
Experimental (E - dots) vs. theoretical (T - line) I-V curves for different irradiances.

The parameters of the OPV cell, are shown in Table 1, and the equivalent circuit (Figure 4a) is shown in Table 2 for the different intensities of incident radiation. The five parameters (*I*_1_, *I_S_*, *n*, *R_s_* and *R_p_*) were obtained by applying a numerical least squares curve fitting technique to the I-V curves of the cells under various light conditions [51] (Figure 7) and based on the equivalent circuit Equation (4) [52]. Thus, in order to observe the accuracy of the resulting theoretical electrical model, the I-V curves of this circuit model, obtained using an electrical simulation program, were plotted for the different irradiances (as shown in Figure 9), and compared with the original I-V experimental data (presented in Table 1).

From Table 1 and Table 2 we can conclude that the output current was reduced by reducing the input radiance, which led to a strong reduction in the output power. The *FF* slightly increased and the OPV cells performance was better for low input radiance.

Using (6), we have obtained that the capacitance *C*, in parallel with the equivalent OPV model was 37.5 nF. For this calculation we have considered a dielectric constant (*K_s_*) for the semiconductor of 3 nm and a thickness (*d*) of 170 nm (thickness of the active layer measured with a profilometer).

## 3. Integrated CMOS Test Circuit

**Figure 10:**
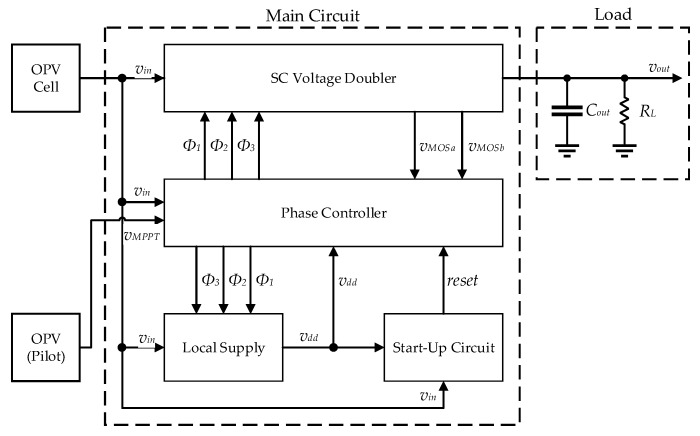
Block diagram of the power management unit (PMU).

The OPV cells studied in this work were connected to an integrated CMOS DC-D SC switch capacitor (SC) converter system with MPPT capability, designed and built using a 0.13 µm CMOS technology [53]. The block diagram of this system is shown in Figure 10.

The SC voltage doubler, local supply, start up, and phase controller blocks were integrated into a CMOS silicon die with a layout area of less than 1 mm^2^.

### 3.1. Switched-Capacitor Voltage Doubler with Charge Reuse

**Figure 11:**
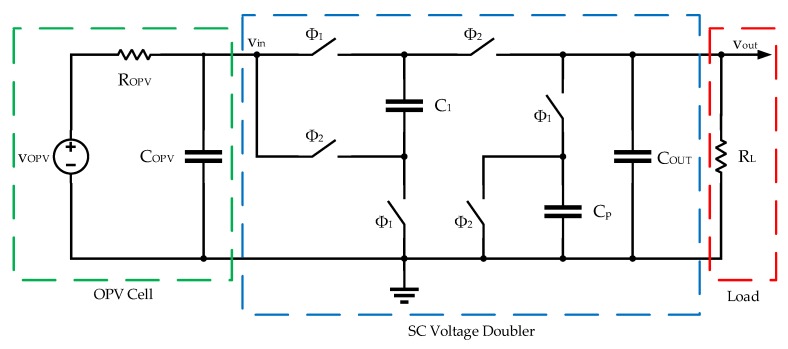
Simplified schematic of the voltage doubler.

A switched capacitor-based converter was chosen to reduce the cost and volume of the overall system by avoiding an external inductor, usually required by inductive switch mode DC–DC converters. A simplified schematic of the step-up converter circuit is depicted in Figure 11.

This schematic also includes a switched parasitic capacitor (*C_p_*), representing the load created by the operation of the phase controller circuit and clock drivers, shown in Figure 11. This controller circuit was responsible for producing the clock phases *ϕ*_1_, *ϕ*_2_, and *ϕ*_3_. Capacitor *C_OPV_* represents the parasitic capacitance of the PV cell, which has been linearized, and is represented by its Thévenin equivalent circuit (*v_S_* and *R_S_*).

The basic principle of operation of this circuit relies of the fact that, during *ϕ*_1_, *C*_1_ is connected in parallel with *v_in_* and charged to this voltage value, whereas in *ϕ*_2_, *C*_1_ is placed in series with *v_in_*, leading to an output voltage (*v_out_*) ideally two times the input.

**Figure 12:**
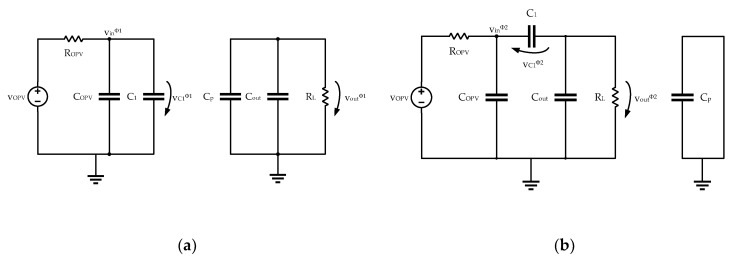
(**a**) Circuit configuration in phase *ϕ*_1_. (**b**) Circuit configuration in phase *ϕ*_2_.

From the analysis of the two configurations of the circuit of Figure 11 (shown in Figure 12), in phases *ϕ*_1_ and *ϕ*_2_ it is possible to write the equations for the conservation of the charge of the circuit capacitors. These equations are obtained considering that *T_CLK_* >> *R_ON_* × *C*_1_ (the switch resistance is negligible) and that *T_CLK_* << *R_OPV_* × *C_OPV_* and *T_CLK_* << *R_L_* × *C_out_*. A detailed theoretical analysis showing how these equations are derived, can be found in [53].

From the charge conservation equations, it is possible to obtain the expressions for the steady-state input and output voltages of the circuit [53] considering an OPV at input:

(9) $$V_{in}=\frac{T_{CLK}(4(C_{out}C_{p}+C_{1}\left(C_{out}+C_{p})\right)R_{L}+\left(C_{1}+4C_{out}+C_{p}\right)T_{CLK})v_{OPV}}{16C_{1}C_{out}C_{p}R_{L}R_{OPV}+4\left(C_{out}C_{p}R_{L}+C_{1}\left(C_{out}+C_{p}\right)R_{L}+C_{1}\left(4C_{out}+C_{p}\right)R_{OPV}\right)T_{CLK}+\left(C_{1}+4C_{out}+C_{p}\right)T_{CLK}^{2}}$$

(10) $$V_{out}=\frac{2C_{1}\left(4C_{o}R_{L}-T_{CLK}\right)T_{CLK}v_{OPV}}{16C_{1}C_{out}C_{p}R_{L}R_{OPV}+4\left(C_{out}C_{p}R_{L}+C_{1}\left(C_{out}+C_{p}\right)R_{L}+C_{1}\left(4C_{out}+C_{p}\right)R_{OPV}\right)T_{CLK}+\left(C_{1}+4C_{out}+C_{p}\right)T_{CLK}^{2}}$$

**Figure 13:**
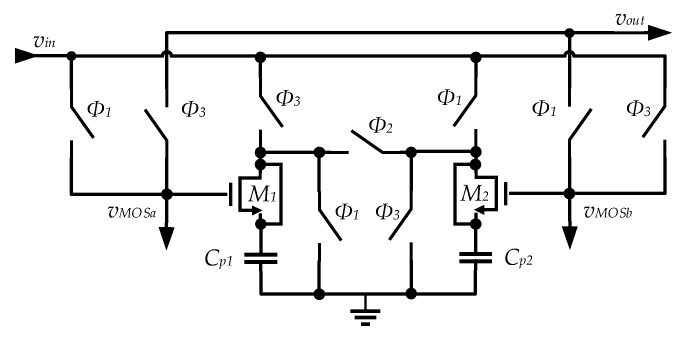
Schematic of the step-up doubler switch capacitor (SC) converter, using MOS capacitors with charge reuse.

To minimize the area, Metal-Oxide-Semiconductor (MOS) transistor capacitors were used that have the largest available capacitance per unit area in the selected CMOS technology, which resulted in a large bottom plate parasitic capacitance, charged to *v_in_* during phase *ϕ*_1_ and discharged during phase *ϕ*_2_. This lowered the efficiency of the circuit and, to improve it, it was necessary to reduce the amount of charge lost through the bottom plate parasitic capacitance. This was done by splitting the capacitance in two and duplicating the circuit. Considering now as *ϕ*_3_ the old *ϕ*_2_ phase, a third phase (*ϕ*_2_), was introduced between *ϕ*_1_ and *ϕ*_3_. During this new phase *ϕ*_2_, the bottom plate nodes of the two half-circuits were connected resulting in charge redistribution between the two parasitic capacitances [53]. This circuit is depicted in Figure 13. Because one of these capacitances was charged to *v_in_*, while the other was completely discharged, when they were connected, the charge was equally divided between them, and each capacitor had half of the charge of the capacitor that was firstly charged to *v_in_*. Either in phase *ϕ*_1_ or in phase *ϕ*_3_, the parasitic capacitor connected to the input will already be pre-charged to half of its final charge value, thus requiring only half of the charge from the input source.

### 3.2. Other Blocks

The clock phases that control the SC voltage doubler had a frequency that maximized the power transfer from the PV cells. Since this frequency changed with the light intensity and temperature, the controller circuit producing these clock phases should use an MPPT method in order to continuously adjust the clock frequency value. The fractional open circuit voltage (Fractional V_OC_) method ([22]) was chosen, because of its simplicity. This method explores the intrinsic characteristic of PV cells, in which there is a proportionality factor (*k*) between their open circuit voltage and the voltage at which the maximum power point (MPP) occurs (*v_MPP_*). This factor must be determined beforehand, by studying the PV cell behavior under several conditions of illumination and temperature.

**Figure 14:**
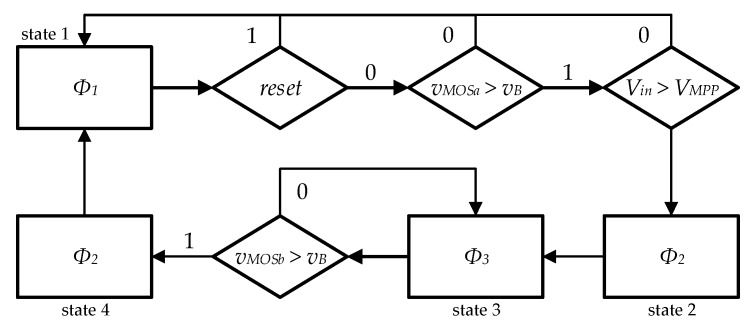
State diagram of the algorithm implemented by the state machine.

Pilot PV cells in open circuit (unloaded) were used to measure the open circuit voltage. The optimum voltage of the unloaded PV cell (*v_MPP_*) was determined by multiplying the open circuit reference pilot voltage by *k*, using a resistive divider. The pilot PV cells can be smaller than the main PV cells and they must have the same temperature and illumination as the main cells, for the fractional V_OC_ method to accurately track the MPP voltage. The state diagram that represents the MPPT algorithm, the generation of the three clock phases, and the conditions that must be met to go from one state to the next, are shown in Figure 14.

Since this is an energy harvesting system, it must have its own power supply for the controller circuit that generates the clock signals. The main output voltage cannot be used to power the clock generation circuit, because during the start-up of the system will remain close to 0 V for a long time, if there is a large capacitance at the output. The solution was to create a supply voltage, independent from the output voltage. This allowed the system to start-up even if the large output capacitor was charged from 0 V.

This local supply block was a smaller SC voltage doubler circuit, controlled by the same phase signals, and was used to create a local supply voltage in an internal decoupling capacitor. This circuit was a replica of the main SC circuit, but, with its capacitors and switches, was scaled down to a fraction of the size of those in the main SC circuit.

Since the local supply voltage was initially 0 V, a start-up circuit was required to convert the input node to the output node of the local power supply. As soon the capacitor was charged to a voltage value enough for the phase generator circuit to start working, this connection was removed, and the circuit started its normal operation. The start-up circuit also provided a reset signal for the phase generator circuit, to guarantee that this circuit started working in state 1 (as shown in Figure 14).

## 4. Test Circuit and Measurement Results

To study the performance of the circuit proposed in [53] considering OPVs as an energy source, it was necessary to readjust the parameters of the PMU to reach maximum efficiency. Since the control system was implemented from the fractional *V_OC_* algorithm, it was required, based on the power curve of the OPV cell, to find the voltage that corresponded to the maximum power value made available by the cell. Based on the characterization of the OPV cells presented in Table 2, and the power curves (Figure 8), the performance of the system for different levels of irradiance with a load of 9.9 kΩ is shown in Table 3. In Table 1 the value of the *k* factor for the cell was obtained for different radiation levels and it varied between 0.65 and 0.83. In order to set MPPT algorithm, an average of the k values, for Table 1, was used in the resistive divider of the CMOS DC–DC converter.

**Figure 15:**
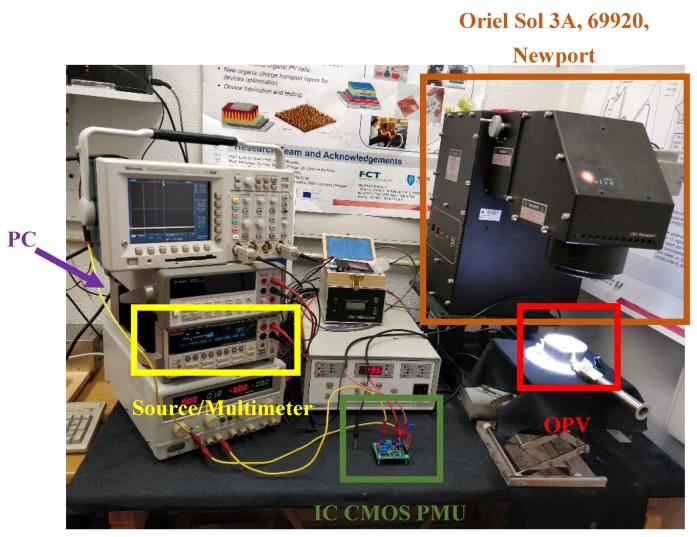
Test setup.

**Figure 16:**
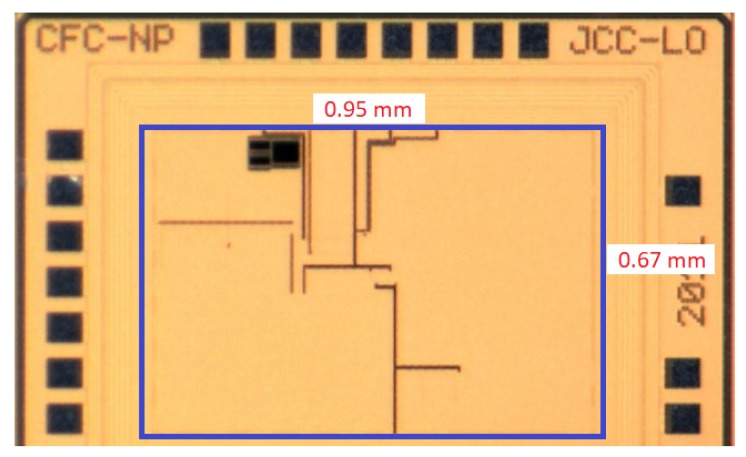
Circuit die micro-photograph.

Due to the inert atmosphere chamber used in the tests of the OPV cells, it was only possible to test two connection configurations, namely with the pixel P_1_ as the main cell and the pixel P_2_ as the pilot cell (Config. 1), and with the two pixels in parallel connected to the input of the circuit (*V_in_*) (Config. 2). In this last configuration, the pilot cell open circuit voltage (*V_OC_*) required for the MPPT algorithm was replaced by an external voltage of 1.2 V. Considering that the two pixels shared a common substrate, the increase of the output power was achieved by connecting them in parallel. Yet, since the output was limited to 0.6 to 0.7 V, the IC CMOS circuit, besides the MPTT, needed to double the output voltage, in order to reach the required 1.2 V. The output power was 0.14 mW, for a low power IoT node, due to limitations in the available CMOS circuit die area. However, this value can be easily increased by using higher area capacitors. The test setup is shown in Figure 15 and circuit die photo (0.95 × 0.67 mm^2^) is shown in Figure 16.

**Figure 17:**
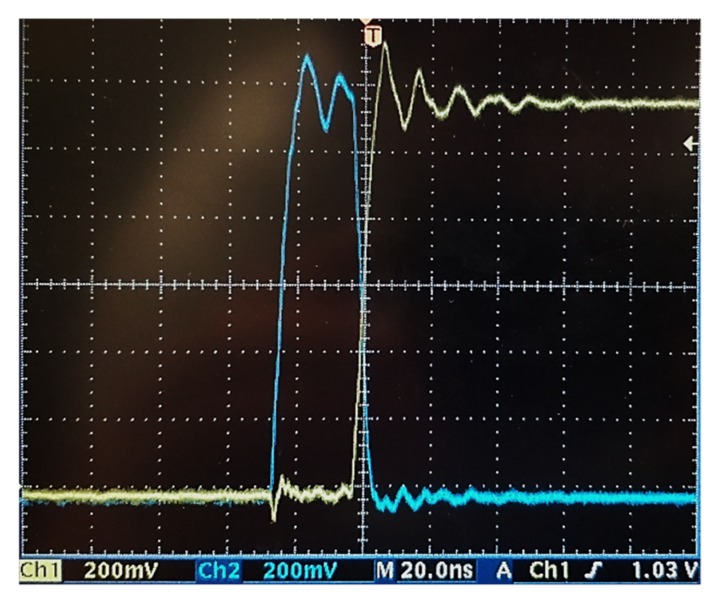
Waveform of phases *Φ*_1_ and *Φ*_2_ for the irradiance of 86.25 mW/cm^2^.

**Figure 18:**
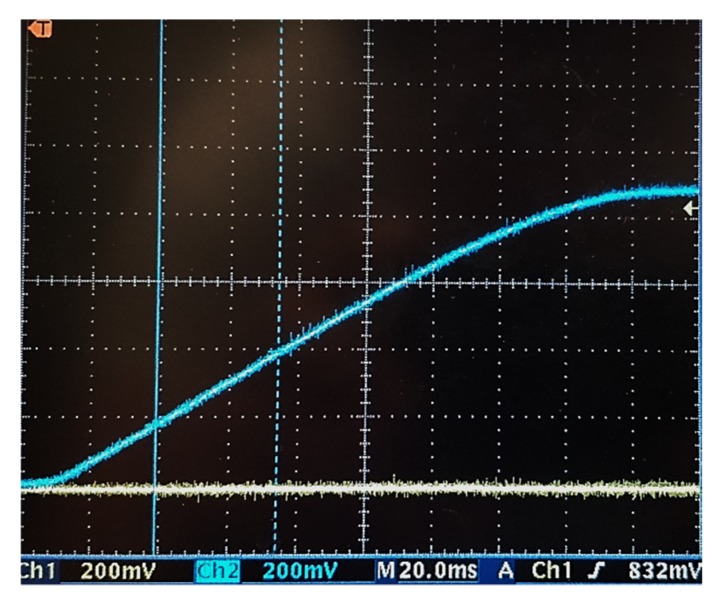
Starting the circuit for the irradiance of 26.21 mW/cm^2^.

In order to validate the correct functioning of the circuit, the waveform of *Φ*_1_ and *Φ*_2_, are shown in Figure 17, channel 1 (Ch1) and channel 2 (Ch2), respectively. The frequency of the clocks can be found in Table 4. Figure 18 shows the start-up of the circuit, for the irradiance level of 26 mW/cm^2^. The above confirms that the control module and the PMU startup worked correctly.

Since the current density of the organic photovoltaic cells was approximately 13 mA/cm^2^, for the maximum irradiance value, it was verified that when the irradiance had decreased, i.e., when the current density is low, the frequencies of *Φ*_1_ and *Φ*_2_ approximate the values for which the system is scaled. Therefore, it is possible to identify the optimal point of operation of the circuit, minimizing the PMU power dissipated by the control module, while the remaining power was delivered to the load.

**Figure 19:**
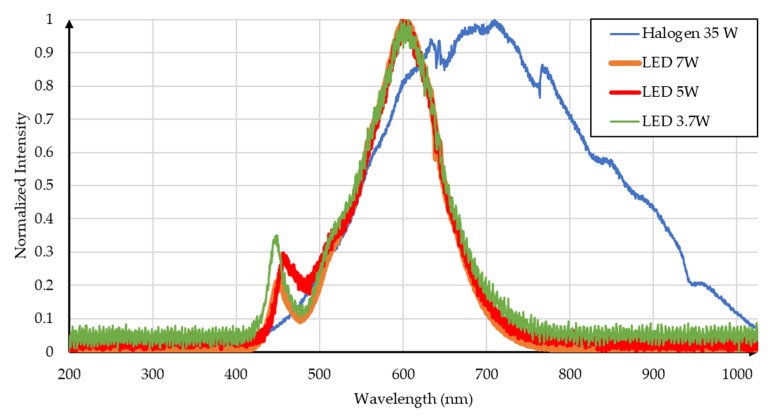
Light spectrum of lamps.

Table 5 shows the technical characteristics of the lamps used. Considering that these characteristics were different from those of the solar simulator with which the first tests were carried out, the light spectrum is also expected to be different. Thus, the standardized luminous spectra of the four lamps considered in this test are shown in Figure 19 for the irradiance described in Table 5.

**Figure 20:**
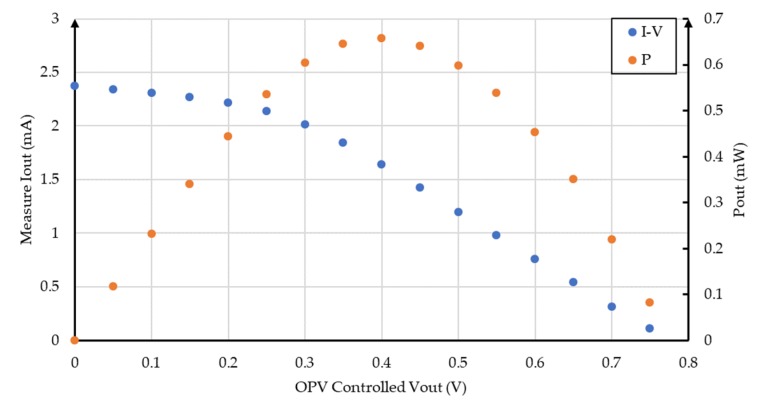
I-V and power curves for the OPV used in the second phase of tests.

The current–voltage and power curves are shown in Figure 20. The short-circuit current of this OPV was 2.27 mA, the open-circuit voltage was 750 mV, and for the maximum power value (660 μW). The voltage value, i.e., the voltage value where the maximum power required for the MPPT algorithm was 400 mV.

The lamps were located at three different (heights) from the OPVs: 0.45 m, 0.26 m, and 0.11 m. Using the Newport 91150V calibration cell, the four lamps were irradiated at different heights, as shown in Table 6.

**Figure 21:**
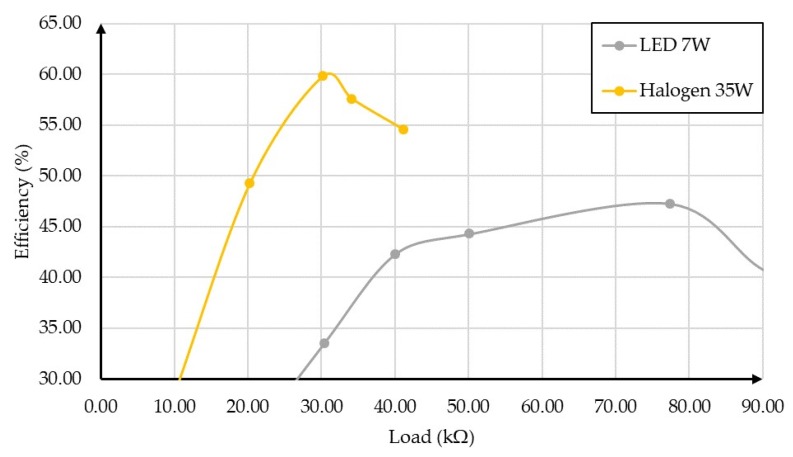
Efficiency as a function of the load for the height of 0.45 m.

**Figure 22:**
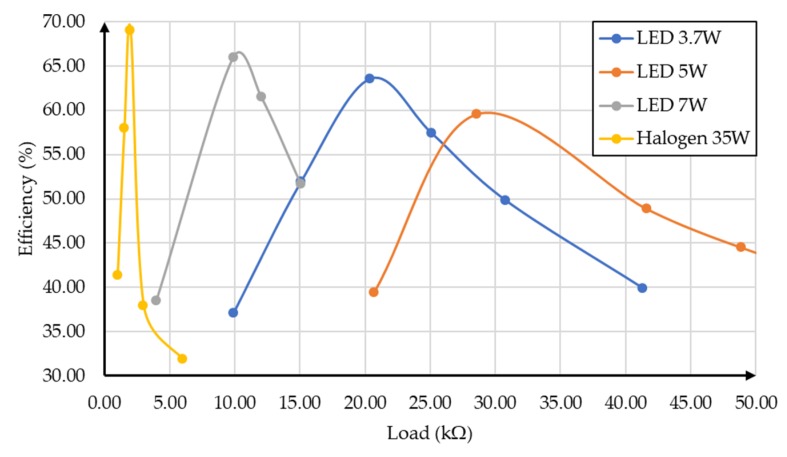
Efficiency as a function of the load for the height of 0.11 m for different light sources.

The maximum efficiency value of the PMU, for each illumination value, was determined by varying the PMU load. Due to the low irradiation power of the 5 W and 3.7 W lamps, the proposed PMU worked only for the minimum height considered (0.11 m). Figure 21 and Figure 22 represent the efficiency as a function of the load of the architecture for the heights of 0.45 m and 0.11 m, respectively, for each lamp. It was not possible to perform the comparison for the height of 0.26 m.

Observing the results in Figure 21 and Figure 22, we can see that the circuit presented maximum efficiency with the 35 W halogen lamp. However, it is necessary to consider the supercharging of the cell in the situation whose height to the work plane is 0.11 m. In this case, the variation of the efficiency as a function of the resistance was very narrow (around 2 kΩ).

In the first set of OPVs, it was verified that the efficiency of the PMU proposed reached 75.76% with an irradiance of 9.52 mW/m^2^ provided by the radiation simulator and a load of 9.9 kΩ (P_out_ = 0.14 mW). A test setup was used with four indoor lamps, each lamp being placed at three levels of height. A maximum efficiency of 65.98% was achieved for the 7 W LED, with an irradiance of 19.31 mW/cm^2^ and a load of 9.9 kΩ (P_out_ = 103.43 μW). Although the efficiency obtained by the remaining lamps were lower, it was above 45%.

When comparing the results with the state-of-the-art harvesters (Table 7), it was noted that the presented PMU had unique features in terms of efficiency and in its construction and implementation. Being the control of this architecture based on a MPPT algorithm, it was guaranteed that the energy provided by the OPV was optimized to the PMU load.

The typical power consumption for IoT nodes is in the order of tens mW, as shown in [54] (Table 4). Furthermore, recently, there have been several papers that have used a SC fully integrated circuit with a lower pout than the proposed PMU in this paper [55].

## 5. Conclusions

In this work, we present a proof of concept that highlights the possibility for the integration of organic photovoltaic cells and a CMOS PMU circuit for indoor light harvesting. The OPV cells were connected to an ultra-low power, low area, and low cost, fully integrated CMOS circuit prototype, designed in a 130 nm process, which used a quasi-MPPT algorithm to maximize the efficiency.

It was shown that the organic cells can operate, combined with a CMOS PMU circuit, in the presence of different indoor light lamps: 35 W halogen, 3.5 W LED, 5 W LED, and 7 W LED. They were irradiated by the light of these lamps from three different heights (0.45 m, 0.26 m, and 0.11 m). For 0.45 m, the maximum efficiency was 60% (35 W halogen) and 45% for 7 W LED. For 0.11 m, they showed a maximum efficiency between 60% (5 W LED) and 70% (35 W halogen).

It was verified that the integrated CMOS circuit presented a maximum efficiency above 75% when the OPV cells were illuminated by the solar simulator, which makes this PMU energy system a promising proof of concept for energy harvesting of organic photovoltaic cells. The results presented in this paper can be scaled up by using a larger OPV and a PMU system with large capacitors in order to be able to supply IoT nodes with higher power requirements.

## Figures and Tables

**Table 1:** Experimental electrical characteristics of an OPV cell.

Irradiance (mW/cm²)	|*I_sc_*| (mA)	*V_oc_* (V)	|*I_mp_*| (mA)	*V_mp_* (V)	*P_max_* (mW)	*k*	*FF*
86.25	3.13	0.77	2.61	0.50	1.31	0.65	0.54
66.34	2.10	0.76	1.66	0.53	0.87	0.69	0.55
46.12	1.42	0.75	1.18	0.55	0.65	0.73	0.61
26.21	0.74	0.73	0.63	0.58	0.36	0.79	0.67
9.52	0.07	0.66	0.06	0.55	0.03	0.83	0.74

**Table 2:** Parameters of the equivalent circuit of an OPV for different irradiances.

Irradiance (mW/cm²)	*I*_1_ (mA)	*I_S_* (pA)	*n*	*R_s_* (Ω)	*R_p_* (kΩ)
86.25	3.23	342.71	1.89	63.93	2.18
66.34	2.20	145.68	1.81	80.88	2.02
46.12	1.48	0.35	1.33	87.47	2.90
26.21	0.76	0.01	1.16	98.91	6.83
9.52	0.07	0.01	1.14	106.13	76.43

**Table 3:** Measured performance of the system proposed in [19] with OPVs.

Config.	Irradiance (mW/cm²)	*V_in_* (V)	*V_mppt_* (V)	*k*	*I_in_* (mA)	*P_in_* (mW)	*V_out_* (V)	*I_out_* (mA)	*P_out_* (mW)	*η* (%)
**1**	86.25	0.65	0.62	0.81	0.43	0.28	1.20	0.11	0.13	47.23
	66.34	0.66	0.63	0.83	0.35	0.23	1.20	0.12	0.14	62.79
	46.12	0.65	0.63	0.84	0.32	0.21	1.19	0.12	0.14	68.92
	26.21	0.62	0.60	0.82	0.43	0.27	1.15	0.12	0.14	51.49
	9.52	0.57	0.51	0.77	0.40	0.23	0.92	0.09	0.08	36.08
**2**	86.25	0.68	0.50	0.65	0.66	0.45	1.14	0.11	0.13	27.93
	66.34	0.69	0.52	0.69	0.63	0.43	1.15	0.11	0.13	29.25
	46.12	0.67	0.55	0.74	0.58	0.39	1.15	0.11	0.13	32.43
	26.21	0.65	0.55	0.76	0.48	0.31	1.17	0.12	0.14	45.05
	9.52	0.63	0.55	0.84	0.29	0.18	1.15	0.12	0.14	75.76

**Table 4:** Frequency of clock *Φ*_1_ e *Φ*_2_.

Irradiance (mW/cm²)	*Φ*_1_ (MHz)	*Φ*_2_ (MHz)
86.25	1.89	7.66
66.34	1.98	7.82
46.12	1.81	7.45
26.21	2.34	8.48
9.52	4.46	16.33

**Table 5:** Technical characteristics of the lamps used.

Lamp	Color Temperature (K)	Irradiance (mW/cm²)
Halogen 35W	3000	23.41
LED 7W	3000	3.34
LED 5W	3000	0.71
LED 3,7W	2800	0.67

**Table 6:** Irradiance (mW/cm^2^) of the different lamps for the three heights.

Lamp	0.45 m	0.26 m	0.11 m
Halogen 35W	6.30	32.50	119.30
LED 7W	1.28	3.73	19.31
LED 5W	-	0.86	2.76
LED 3.7W	0.18	0.44	1.56

**Table 7:** Performance comparison with state-of-the-art power management units.

Article	[56]	[57]	[58]	[59]	[60]	[61]	[61]	This Work
Technology	0.35 μm CMOS	0.25 μm CMOS	0.18 μm CMOS	0.13 μm CMOS	0.13 μm CMOS	SMT	SMT	0.13 μm CMOS
Year	2010	2011	2012	2014	2016	2017	2017	2018
Vin (V)	0.025	0.5–2	0.12	0.6	0.4	0.65	0.65	0.63
Vout (V)	1.8	0–5	1.2	1.2	2.4	1.93	2.7	1.15
Area (mm^2^)	1.7	N/A	0.273	0.31	0.204	228	215	0.6
Freq. (MHz)	N/A	0.1	1–5	0.1–0.3	10	1.2	1.2	0.1–0.3
Efficiency (%)	58	70	30	70.3	N/A	N/A	N/A	75.76
